# Do we need to consider motion artefacts other than swallowing in carotid artery imaging?

**DOI:** 10.1186/1532-429X-11-S1-P25

**Published:** 2009-01-28

**Authors:** Cheuk F Chan, Peter Gatehouse, Dudley J Pennell, David N Firmin

**Affiliations:** Royal Brompton Hospital, London, UK

**Keywords:** Carotid Artery, Wall Imaging, Carotid Artery Wall, Respiratory Movement Artefact, Head Support

## Introduction

Recent advances in carotid artery wall imaging have seen a transition from 2D to more efficient 3D imaging [1] without sacrificing image quality [2]. A disadvantage of 3D scans is their longer time to complete the acquisition, making them more susceptible to motion artefacts, particularly swallowing [3] and bulk head motion during the long 3D acquisition time. Respiratory movement artefact is controversial [4], but Boussel [5] demonstrated its detrimental effect on carotid wall imaging, using real-time transaxial cines. However, during quiet supine respiration, breathing is predominantly diaphragmatic resulting in the greatest carotid movement in the head-foot direction. We used a novel high temporal resolution interleaved approach to study carotid artery movement in all directions, over the typical 3D scan duration, for a true representation of the potential problem for 3D imaging.

## Purpose

This study aimed to measure bulk respiratory and swallowing motion affecting carotid artery imaging and investigates optimal head support for carotid work.

## Methods

**Figure 1:**
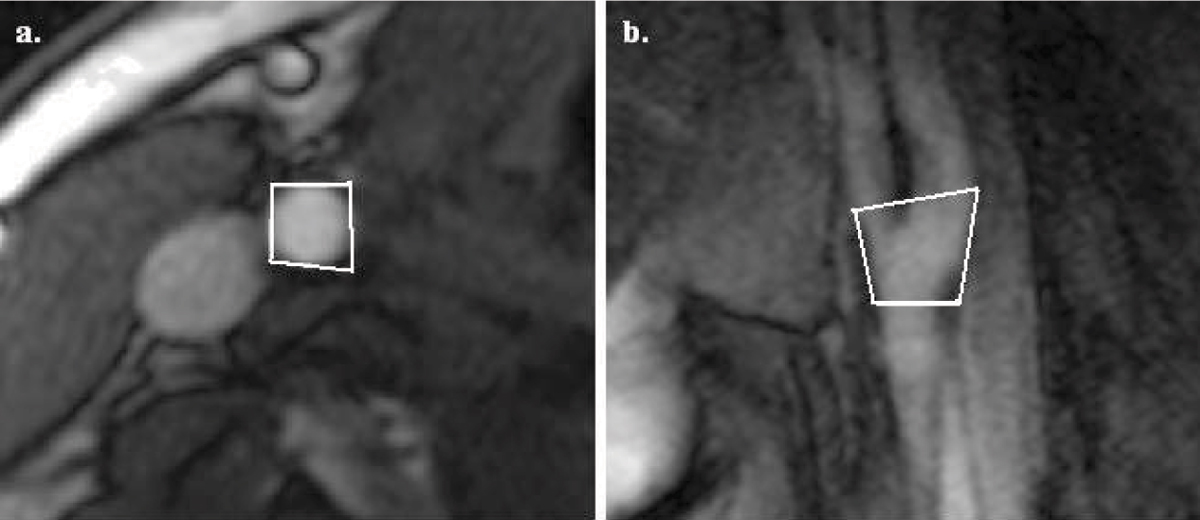
**(a) Regions of interest are drawn around the common carotid artery in the transverse plane and (b) in the oblique-sagittal plane allowing for ROI automatic tracking using MATLAB after image acquisition**.

Nineteen healthy volunteers were scanned at 1.5 T (Siemens Avanto) with phased array carotid coils (Machnet). They had high-resolution bSSFP scans of the right carotid artery in the oblique-sagittal plane centered about the bifurcation and in the transverse plane to measure movement in the Head-Foot, Antero-Posterior and Left-Right directions. Each volunteer was scanned twice: with a vac-lok™ fixation pillow (CIVCO Medical Solutions) followed by a standard Siemens foam head support. Subjects were all asked ''to lie still and relax'' during scans. For 500 cardiac cycles, ECG-triggered diastolic oblique-sagittal and transverse single-shot bSSFP images were alternately acquired (one image/cardiac cycle) with pixel size 0.6 × 0.6 × 6 mm, ETL 1, flip angle 70°, TR 395 ms, TE 1.6 ms. Image post-processing using 2D-correlation tracked an ROI with MATLAB^®^ (The MathWorks) (figure 1). For this, image pixels were 2D-interpolated to 0.31 × 0.31 mm.

## Results

**Figure 2:**
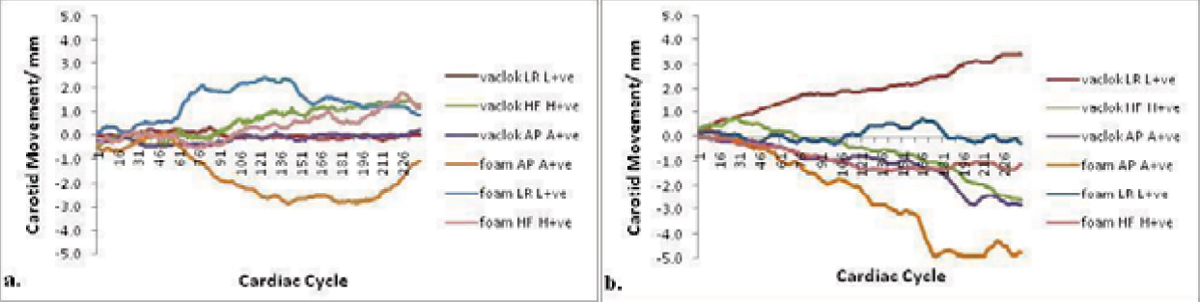
**(a) Demonstrates the "drift" phenomenon in all three perpendicular directions with both types of head support after smoothing**. The "drift" appears to increase as the scan progresses but then seems to decrease towards the end. This could be due to the individual falling asleep during the scan but becoming more alert towards the end. Whereas, in **(b)**, there is a gradual "drift" away from baseline until the end of the scan. In this example, the phenomenon may be explained by increased muscle tension at the start; individuals are generally tense and anxious prior to the scan but once the scan has commenced, they start to relax and so muscle tension decreases resulting in the observed "drift".

There was no obvious bulk head motion during scans with either pillow. In 30/38 scans, there was "drift" of the carotid artery compared to the baseline reference image with no clear pattern. "Drift" was seen in all three directions with both types of head support (figure 2).

During a swallow event, there was variable movement of carotid artery in all 3 directions. To measure swallowing motion the position measurements were compared between smoothed (20-cycle running average, swallows filtered out) and unsmoothed motion traces. Examining all the scans, the greatest movement was predominantly in the head-foot direction with average movement of 3.5 mm (SD 1.8, range 1.6–6.2 mm). Left-Right movement was greater in comparison to Antero-Posterior, 2.8 mm (SD 1.4, range 0.9–6.2 mm) and 1.7 mm (SD 1.5, range 0.6–5.1 mm), respectively.

The amount of movement during quiet respiration was calculated between smoothed and unsmoothed traces excluding swallow events. The largest average movement was in the head-foot direction 1.4 mm (SD 0.6, range 0.6–3.2 mm), with substantially less movement in the LR 0.9 mm (SD 0.5, range 0.3–2.4 mm) and AP 0.5 mm (SD 0.3, range 0.1–1.1 mm).

Although no obvious difference in head motion was measured between the different head supports, individuals reported that the vac-lok™ pillow was more comfortable and offered greater neck support.

## Conclusion

We have shown that respiratory motion previously regarded as insignificant may be large enough to contribute to blurring and ghosting of 3D carotid images. Involuntary head motion even in motivated volunteers during 3D imaging times is clearly an issue for 3D imaging reliability, as is swallowing motion in some subjects. These motion artefacts can corrupt the entire 3D sequence and affect wall imaging and plaque characterisation.
